# Role of Telemedicine in Improving Access to Healthcare

**DOI:** 10.31661/gmj.v13i.3214

**Published:** 2024-05-08

**Authors:** Afsaneh Halili, Fatemeh Zarepour, Fatemeh Kyani, Negin Madadzadeh, Arezoo Yousefi, Fateme Shariati Far

**Affiliations:** ^1^ Department of Critical Care Nursing, Isfahan University of Medical Science, Isfahan, Iran; ^2^ Department of Nursing, School of Nursing and Midwifery, Ahvaz Jundishapur University of Medical Sciences, Ahvaz, Iran; ^3^ Department of Midwifery, Astara Branch, Islamic Azad University, Astar, Iran; ^4^ Department of Nursing, Community Health Research Center, Isfahan (Khorasgan) Branch, Islamic Azad University, Isfahan, Iran; ^5^ Department of Nursing, Student Research Center, School of Nursing and Midwifery, Isfahan University of Medical Sciences, Internal-surgery Trend, Iran

**Keywords:** Telemedicine, Technology, Healthcare

## Dear Editor,

**Table:** Table1. Most Popular Telemedicine Services

**Services**	**Description (s) **	**Platforms**
**Amwell**	-It offers online doctor visits, providing 24/7 access to primary care physicians and specialists. -Patients can receive medical advice, prescriptions, and follow-up care via video consultations.	Web, iOS, Android
**Teladoc**	-It is a telemedicine platform that connects patients to licensed healthcare providers who can diagnose, treat, and prescribe medications for various non-emergency medical conditions. -It covers a broad range of specialties, including general medicine, mental health, and dermatology.	Web, iOS, Android
**Doctor On Demand **	-It provides video consultations with doctors, therapists, and psychiatrists. -It offers services for general medical concerns, mental health support, and chronic condition management. -Appointments can be scheduled or accessed on-demand in some regions	Web, iOS, Android
**MDLIVE**	-It allows patients to consult with board-certified doctors and pediatricians via video or phone calls. -It covers a wide range of medical issues, including routine illnesses, dermatology, and behavioral health. -The platform facilitates prescription delivery to local pharmacies.	Web, iOS, Android
**PlushCare**	-It offers virtual visits with primary care physicians and specialists. -Patients can receive consultations, diagnoses, prescriptions, and lab testing referrals. -The platform features same-day appointments and the ability to communicate with providers via secure messaging.	Web, iOS, Android
**LiveHealth Online **	-It connects patients with healthcare professionals for non-emergency medical conditions. -It enables video visits with doctors, pediatricians, therapists, and psychiatrists. -The service includes options for prescription refills and therapist follow-ups.	Web, iOS, Android

Telemedicine, or the use of technology to provide remote medical care, has the potential to revolutionize the method of delivering healthcare services, particularly in areas where access to medical care is limited [1]. Currently, telemedicine systems are gaining popularity in the world, each of which has its own advantages and disadvantages (Table-1). We have tried to point out the most important advantages and problems of these systems and specify the possible solutions.

One of the key benefits of telemedicine is that it can help to overcome geographical barriers to healthcare [2]. In rural or remote areas, for instance, patients may need to travel long distances to access medical care, which can be both time-consuming and expensive [3]. Hence, telemedicine can overcome this issue by allowing patients to consult with healthcare providers remotely, using video conferencing, phone calls, or other digital communication tools [4].

Another benefit of telemedicine is that it can help to improve access to specialist care [5]. In many areas, there may be a lack of specialists in certain fields, such as cardiology or neurology. Telemedicine can help to connect patients with these specialists, regardless of their location, allowing them to receive the care they need from long distances [6]. Also, telemedicine can help to improve access to healthcare for vulnerable populations, such as the elderly or those with disabilities [7]. These patients may face challenges in accessing medical care due to movement disorders or other health concerns [8]. Hence, telemedicine could overcome these barriers by allowing patients to consult with healthcare providers from the comfort of their own homes.

Reducing the cost of healthcare is one of the most significant advantages of telemedicine [9]. Indeed, by providing remote medical care, the need for patients to travel to medical facilities. Additionally, telemedicine decreases the need for hospitalizations and emergency room visits, which can be costly for both patients and healthcare providers [10]. In addition, it can improve patient outcomes by providing more timely access to medical care [11]. Indeed, in many cases, patients may delay seeking medical care due to the inconvenience or expense of traveling to a medical facility. With telemedicine, patients can consult with healthcare providers more quickly and easily, allowing them to receive the care they need before their condition worsens [12].

Another benefit of telemedicine is enhanced patient engagement and satisfaction. By providing remote medical care, telemedicine can help to make healthcare more convenient and accessible for patients, which can improve their overall experience [13]. Additionally, telemedicine can help to improve patient education and self-management, allowing patients to take a more active role in their own healthcare [14].

Telemedicine may provide healthcare delivery in emergencies. In the event of a natural disaster or other emergency, telemedicine could provide medical care to patients who may not be able to access medical facilities due to road closures or other issues [15].

Despite the many benefits of telemedicine, some challenges must be addressed. One of the most important challenges is ensuring that patients have access to the necessary technology [16]. In many areas, patients may not have access to high-speed internet or other digital communication tools, which can limit their ability to use telemedicine [17].

Another challenge is ensuring that healthcare providers are trained in the use of telemedicine. Many healthcare providers may not be familiar with the technology or may not have the necessary equipment to provide remote medical care [18]. Additionally, healthcare providers may need to be trained in new skills, such as conducting virtual exams or interpreting remote diagnostic tests [19].

However, telemedicine cannot replace all in-person consultations, as certain medical conditions require physical examination or procedures that cannot be performed remotely [20]. It is crucial to make a balance between telemedicine and in-person care to provide comprehensive and holistic healthcare services.

Ethical considerations also arise in telemedicine, including patient privacy and medical data security [21]. Robust telemedicine platforms must adhere to strict data protection regulations and ensure secure transmission and storage of patient information.

Despite these challenges, telemedicine has immense potential to revolutionize healthcare delivery and improve access to care [22]. Continued research and development are necessary to optimize telemedicine platforms and address existing limitations. Also, policy development is crucial to ensure reimbursement for telemedicine services, remove regulatory barriers, and establish standards to ensure safe and effective practice.

In conclusion, telemedicine has significant implications for improving access to healthcare by expanding access for underserved populations, facilitating timely care, and enhancing convenience. While challenges exist, addressing technological barriers and ethical considerations will enable the widespread adoption of telemedicine. With further research, policy development, and collaboration between healthcare providers and technology experts, telemedicine can play a transformative role in ensuring equal distribution of access to healthcare for all.

## Conflict of Interest

All the authors declare that there are no conflicts of interest.
